# Behavioral and Transcriptome Profiling of Heterozygous Rab10 Knock-Out Mice

**DOI:** 10.1523/ENEURO.0459-22.2023

**Published:** 2023-05-18

**Authors:** Wyatt Bunner, Jie Wang, Sarah Cohen, Denys Bashtovyy, Rachel Perry, Daniel Shookster, Taylor Landry, Elizabeth M. Harris, Robert Stackman, Tuan D. Tran, Ryohei Yasuda, Erzsebet M. Szatmari

**Affiliations:** ^1^Department of Physical Therapy, East Carolina University, Greenville, NC 27834; ^2^Max Planck Florida Institute for Neuroscience, Jupiter, FL 33458; ^3^Jupiter Life Science Initiative, Florida Atlantic University, Jupiter, FL 33458; ^4^Department of Kinesiology, East Carolina University, NC 27858; ^5^Department of Psychology, East Carolina University, Greenville, NC 27858

**Keywords:** behavior, GTPase, learning and memory, neurodegeneration, NMDA receptors, Rab10

## Abstract

A central question in the field of aging research is to identify the cellular and molecular basis of neuroresilience. One potential candidate is the small GTPase, Rab10. Here, we used Rab10^+/−^ mice to investigate the molecular mechanisms underlying Rab10-mediated neuroresilience. Brain expression analysis of 880 genes involved in neurodegeneration showed that Rab10^+/−^ mice have increased activation of pathways associated with neuronal metabolism, structural integrity, neurotransmission, and neuroplasticity compared with their Rab10^+/+^ littermates. Lower activation was observed for pathways involved in neuroinflammation and aging. We identified and validated several differentially expressed genes (DEGs), including Stx2, Stx1b, Vegfa, and Lrrc25 (downregulated) and Prkaa2, Syt4, and Grin2d (upregulated). Behavioral testing showed that Rab10^+/−^ mice perform better in a hippocampal-dependent spatial task (object in place test), while their performance in a classical conditioning task (trace eyeblink classical conditioning, TECC) was significantly impaired. Therefore, our findings indicate that Rab10 differentially controls the brain circuitry of hippocampal-dependent spatial memory and higher-order behavior that requires intact cortex-hippocampal circuitry. Transcriptome and biochemical characterization of these mice suggest that glutamate ionotropic receptor NMDA type subunit 2D (GRIN2D or GluN2D) is affected by Rab10 signaling. Further work is needed to evaluate whether GRIN2D mediates the behavioral phenotypes of the Rab10^+/−^ mice. We conclude that Rab10^+/−^ mice described here can be a valuable tool to study the mechanisms of resilience in Alzheimer’s disease (AD) model mice and to identify novel therapeutical targets to prevent cognitive decline associated with normal and pathologic aging.

## Significance Statement

Alzheimer’s disease (AD) is characterized by a widespread collapse of neuronal circuits that precedes amyloid plaque deposition and tau pathology. Yet, 30–50% of older individuals, who harbor the anatomic and molecular features of AD, preserve their cognitive abilities, and do not show AD symptoms in their lifetime. It has been suggested that the Rab10 protein is among the mediators of “cognitive resilience” against AD. The focus of this work was to characterize Rab10^+/−^ mice behaviorally and molecularly, and to identify downstream targets of Rab10-dependent neuroresilience. The Rab10^+/−^ mice described here can be used to study cellular and molecular mechanisms of Rab10-dependent resilience in mouse models of AD.

## Introduction

Rab10 is a small monomeric Ras-related GTP-binding protein widely distributed in the intracellular membranes. It is highly conserved from *Caenorhabditis elegans* to humans, with a predicted molecular weight of ∼23 kDa (Lv et al., 2015). Similar to other Rab proteins, Rab10 regulates intracellular trafficking by recruiting effectors and binding proteins (Homma et al., 2021). Among the 60 human Rab GTPases, only 24 Rab proteins are specific to or enriched in neurons (Stenmark and Olkkonen, 2001; Brighouse et al., 2010; Diekmann et al., 2011; D’Adamo et al., 2014; Homma et al., 2021). In the CNS, Rab protein-dependent membrane trafficking (including vesicle biogenesis, sorting, fission, transport, tethering, docking, and fusion) is essential for the development of neuronal asymmetry and the formation of neuronal circuits (Mignogna and D’Adamo, 2018). In particular, Rab10 is a crucial regulator of axonal development, dendritic arborization, and glutamate receptor trafficking during synaptic plasticity (Deng et al., 2014; Taylor et al., 2015; Zou et al., 2015; Mignogna and D’Adamo, 2018; Homma et al., 2021).

As small GTPases, Rab proteins are switched between the guanosine diphosphate (GDP)-bound “inactive” state and the GTP-bound “active” state. Specific guanine nucleotide exchange factors (GEFs) regulate the conversion from “active” to “inactive” state, while GTPase activating proteins (GAPs) control the opposite way (Stenmark, 2009). Activated Rab GTPases interact with diverse downstream effectors to coordinate the intracellular transport of proteins and lipids (Hutagalung and Novick, 2011). In neurons, membrane trafficking is essential to the maintenance of asymmetric morphology and synaptic activity. Several Rab proteins have been implicated in neurodegenerative disorders, including Alzheimer’s disease (AD), Parkinson’s disease (PD), Huntington’s disease, and motor neuron degeneration (Bezprozvanny and Hiesinger, 2013; Kiral et al., 2018).

Although aging is the main risk factor associated with sporadic Alzheimer’s disease, several genetic risk factors have been identified, including rare variants in APP, PLD3, and TREM2 (Guerreiro et al., 2013; Jonsson et al., 2013; Cruchaga et al., 2014) along with a few protective rare variants in APP and CD33 (Jonsson et al., 2012; Griciuc et al., 2013; Medway et al., 2014). A recent whole genome sequencing (WGS) study identified a rare functional variant in Rab10 which confers “resilience” against AD even for high-risk individuals (Ridge et al., 2017; Tavana et al., 2019). Moreover, in a cellular model of AD, silencing of Rab10 reduced Aβ42 production and Aβ42/40 ratio, while Rab10 overexpression had the opposite effect (Ridge et al., 2017). Another line of evidence implicates Rab10 in AD-associated tau pathology. Rab10 was found to be hyperphosphorylated at the T73 residue and co-localized with neurofibrillary tangles (NFTs) in the postmortem human hippocampus (Pavlos and Jahn, 2011). Rab10 hyperphosphorylation at T73 in human AD hippocampus was shown to be mediated by the serine/threonine protein kinase, leucine-rich repeat kinase 2 (LRRK2), leading to the aberrant membrane and vesicle trafficking and progression of neurodegeneration (Yan et al., 2018). In addition, variants in LRKK2 are among the most common genetic risk factors for Parkinson’s disease, the second leading neurodegenerative disorder (Steger et al., 2016; Chen et al., 2017; Z. Liu et al., 2018). Given that several LRKK2 variants increase LRKK2 kinase activity toward Rab10, an emerging new approach for disease modification in Parkinson’s disease is inhibition of the LRRK2-Rab signaling pathway and subsequently restoring normal membrane trafficking and lysosomal activity (Jennings et al., 2022).

Despite the emerging roles of Rab10 in brain function and neurodegeneration (particularly in AD and PD pathophysiology), our mechanistic understanding of Rab10’s functions is still limited. Here, using heterozygous knock-out mice for Rab10 (Rab10^+/−^), we performed cellular, molecular, and behavioral assessments of the effects of a reduced Rab10 level in the mouse brain. We provide evidence that Rab10 differentially regulates spatial memory and higher-order learning. Transcriptome profiling suggests that Rab10 downregulation leads to elevated activation of signaling pathways associated with neuronal metabolism, structural integrity, neurotransmission, and neuroplasticity, as well as reduced activation of neuroinflammatory and aging pathways in Rab10^+/−^ mice. Differentially expressed genes (DEGs) in Rab10^+/−^ mice (downregulated: Stx2, Stx1b, Vegfa, and Lrrc25; upregulated: Prkaa2, Syt4, and Grin2d) are associated with neuronal metabolism, structural integrity, and synaptic transmission. Thus, this study presents novel roles for Rab10 in the brain and identifies potential mediators of neuroresilience provided by a reduced level of Rab10.

## Materials and Methods

### Mice

All mice were housed in the Animal Resource Facilities compliant with the National Institutes of Health *Guide for Care and Use of Laboratory Animals*. Rab10 conditional knock-out (cKO) mice were generated from C57BL/6 mice. A targeting vector for BAC recombineering was generated with two 50 bp homology regions located 265–365 bp upstream of Rab10 exon 2. The two homology regions were upstream and downstream of a frt site-flanked neomycin cassette with a single lox site at the 3′ end. This vector was inserted into a Rab10 locus-containing BAC (RP24-278M14; CHORI) through homologous recombination in *Escherichia coli* (Murphy, 1998; Y. Zhang et al., 1998; Muyrers et al., 1999; Yu et al., 2000), thereby placing a neo cassette and one lox site 265 bp upstream of exon 2. The second 3′ lox site was placed 169 bp downstream of exon 2 by co-integration during the above homologous recombination process of a PCR fragment containing the lox site flanked by 300 bp homology regions. Subsequently, an embryonic stem (ES) cell targeting vector containing the modified Rab10 locus was retrieved through recombineering the BAC into a plasmid vector (PL253; P. Liu et al., 2003). In 129 Sv ES cells (R1; Nagy et al., 1993), homologous targeting of the Rab10 locus was achieved by electroporation of the linearized targeting vector, selection of neo-resistant colonies, and PCR screening for homologous integration of the targeting vector. Correctly modified ES cells were then injected into C57BL/6 blastocysts following standard procedures. Chimeric males were mated with black wild-type (WT) females (C57BL/6) to generate heterozygous transgenic mice and further crossed with Flp mice to remove the neo cassette. The heterozygous Rab10 cKO mice were further crossed with CMV-Cre mice to generate the heterozygous constitutive Rab10 KO mice in all tissues.

In all experiments, the genotype of each mouse was verified by PCR of genomic DNA extracted from the tail before experiments and by Western blotting of brain samples after experiments.

The experimental groups are heterozygous Rab10 cKO X CMV-Cre mice, and controls are the wild-type littermates, ensuring that the mice are from the same parents, living in the same cage and are uniformly affected by any environmental effect.

### Behavioral studies

Behavioral testing included open field (OF), object-in-place (OIP), elevated plus maze (EPM), spontaneous alternation (SA), Rota-Rod (RR), Morris water maze (MWM), trace fear conditioning (FC), and trace eyeblink classical conditioning (TECC).

#### Open field test

Locomotor behavior was measured in 43.2 × 43.2 cm square acrylic open field chambers. Before testing, uniformity of light across the arena was confirmed using a light intensity meter, and the chambers were cleaned with 1% Micro-90 before and between trials. Background white noise (∼72 dB) was used during trials. Mice were placed into the center of the chamber to begin testing, and activity was recorded for 30 min. Data were analyzed in 10 min blocks.

#### Elevated plus maze test

Mice were placed in the center of the plus maze (Med Associates) and allowed to explore the maze for 5 min. Time spent in open and closed arms, number of arm entries, latency to initially enter an open arm, and total distance moved were recorded using EthoVision XT (Noldus Information Technology Inc.). Uniformity in lighting was confirmed across the maze, and the maze was cleaned with 1% Micro-90 before each trial. Background white noise used during trials was ∼72 dB.

#### Trace fear conditioning

In trace fear conditioning, there is a temporal gap during which an association can develop between the termination of the tone (CS, conditioned stimulus) and the onset of the aversive stimulus (US, unconditioned stimulus). During the conditioning, mice gradually acquire an appropriately timed anticipatory conditioned freezing response. The strength and accuracy of this temporally guided fear memory then can be assessed during a subsequent test session. As previously described (G. Zhang and Stackman, 2015) each mouse was allowed to freely explore a conditioning chamber during a 10 min Context A preexposure session. Context A consisted of rectangular chambers (30.5 × 24.1 × 21 cm) constructed of brushed aluminum side walls and clear Plexiglas front, back, and top walls. The chamber floor was constructed of parallel stainless-steel rods (36 rods, 3.2 mm in diameter, placed 7.9 mm apart). Before each trial, the chamber floors were cleaned thoroughly with a 10% ethanol solution then with 1% LiquiNox to remove olfactory cues. Twenty-four hours later, each mouse was returned to Context A. After a 60 s exploration interval (used to establish baseline freezing), a tone (90 dB, 5000 Hz, CS) was presented for 15 s followed by a 30 s stimulus-free interval, and then a 0.5 s, 0.75 mA foot shock (US) was presented. The CS-US pairing was repeated eight times with a 210 s intertrial interval (ITI). Sixty seconds after the final CS-US pairing, each mouse was removed from their chamber and returned to their home cages. Twenty-four hours later, each mouse was tested for freezing to the CS tone in a modified chamber (Context B). Context B chamber consisted of a white Plexiglas floor, a black Plexiglas triangular insert, and several drops of 10% acetic acid on the tray under the floor. Thus, Context B provided an altered floor texture, light intensity, chamber geometry, and odor. Sixty seconds after the introduction of a mouse into Context B, eight unpaired 15 s CSs were presented with an ITI of 210 s. Freezing, a rodent’s conditioned response to a threatening stimulus, was recorded and automatically scored by the Video Freezing software (MED Associates).

#### Morris water maze test

The water maze consisted of a 1.4 m diameter white tank with a 10 cm diameter platform submerged ∼1 cm below the surface of the water. The water was made opaque by the addition of nontoxic white washable paint that made the platform invisible during trials. The temperature of the water was kept at 22–24°C. Visual cues were placed in the testing room around the tank for spatial reference. Before water maze training, mice received a visual platform test where the spatial cues were removed, and the platform was elevated above the surface of the water and marked with a cue, so it could clearly be discerned. Mice were given four trials, and the platform location was changed each trial. Visible platform training served to verify the visual ability of the mice and to ensure that the mice had no deficit that would affect their ability to swim to the platform. For the hidden platform training, mice were given four acquisition trials per day for eight consecutive days. The start location was varied for each trial, and the mice were allowed 60 s to find the platform. Mice were left on the platform for 15 s before removing them from the water maze. If a mouse did not find the platform within 60 s, it was placed on or guided to the platform and kept there for 15 s. Mice were dried after each trial and placed into cages located atop heating pads to reduce opportunity for hypothermia. Measures from the daily four-trial block of acquisition trials (escape latency, cumulative distance, and swim speed) were averaged for analysis. On day 9, mice were given a 60 s probe test during which the platform was not present. Activity and performance were tracked using EthoVision XT (Noldus Information Technology Inc.). Total time spent in each quadrant, total number of entries into the target quadrant, total number of platform crossings, latency to first platform crossing, and average distance to the platform center were recorded from each probe test.

#### Spontaneous alternation test

Mice received two tests, each separated by 3 d. Two mazes were used, each turned in a different configuration to increase novelty to the maze on the second test. Each maze contained three arms with walls made opaque including a start box (17.8 × 7.3 cm) at the base of the start arm (38.1 × 7.3 cm) and adjoined to a central choice area (10.2 × 10.2 cm) with two choice arms (30.5 × 7.3 cm) radiating 180 degrees from the central choice area (forming a “T”). Automatic guillotine doors were installed at the entry of each arm that were controlled by EthoVision. Each test was conducted as follows: a mouse was placed in a start box and the door to the maze subsequently opened, allowing the mouse to enter the maze and explore to the T intersection. Upon reaching the intersection, the mouse chose an arm (free choice trial) and, after three body points had entered that arm, the door closed automatically, detaining the mouse in that arm for a period of 10 s. During the 10 s, a cloth lightly sprayed with 70% ethanol was used to wipe the maze outside the chosen arm to remove possible odor cues. After 10 s, the mouse was placed back in the start box for a second free choice trial, after which the door to that arm again closed, detaining the mouse in that arm until prompt removal. Of the two tests the mouse was given, one allowed the mouse to immediately enter the maze for the second trial (no delay trial) and one kept the mouse in the start box for 60 s before the door opened to allow the mouse to enter the maze for a second trial (delay trial). Groups were balanced for maze, test day, and delay. Alternation success was calculated for each test. If a mouse did not leave the start box to enter the maze after 60 s, it was gently nudged with a cotton swab. The maze was cleaned with 70% ethanol between mice. Background white noise (∼72 dB) was present during trials.

#### Object-in-place memory test

The OIP test was performed as previously described (Szatmari et al., 2021). Briefly, the apparatus consisted of two open-top, high-walled square arenas made of white ABS plastic (each: 37.5 × 37.5 × 50.0 cm). A salient landmark cue (blue plastic tarp, 20.3 × 25.4 cm) was affixed with clear tape to the center of the north wall. Each mouse was habituated to one of the arenas for 10 min/day for two consecutive days. On days 3 and 4, each mouse was returned to the familiar arena that contained 2 novel toy objects (stainless steel cabinet leveling foot attached to a Plexiglas base, 4.2 cm in diameter and 6.0 cm tall; metal spring attached to a Plexiglas base, 2.0 cm in diameter and 4.8 cm tall) for 10 min training sessions. The two objects were positioned on the arena floor 2 cm from the corners on either side of the landmark cue (NW and NE). During the test session 24 h later (day 5), each mouse was given a 5 min test session in the familiar arena, yet one of the toy objects was transferred to the opposing southern corner. The objects, the arena floor, and walls were cleaned with 70% ethanol after each session. All behavioral testing data were digitally acquired by the EthoVision XT software package. Object exploration was scored off-line from the digital video files by experimenters that were blind to the genotype of the mice. Object-in-place memory was inferred from the preference ratio, calculated for each subject by dividing the time spent exploring the familiar object in the novel location by the total time spent exploring both objects. Preference ratios range from 0 to 1, with 0.5 indicating chance performance, a lack of preference for one object location over another, and positive ratios indicating preference for exploring the object in the novel location. During training, mice that did not explore the objects for a minimum of 50 s were excluded from analyses. The data for mice that did not explore the objects for a minimum of 20 s during the test session were also excluded from all analyses.

#### Trace eyeblink classical conditioning

All surgical, testing, and analysis procedures were conducted as previously described in rats (Rufer et al., 2012; J.D. Thomas and Tran, 2012; Tran et al., 2017) and scaled for application in mice.

##### Surgery

Mice were implanted with two size 3T stainless steel recording electrodes (Pyrofuze Corp/Medwire) in the left orbicularis oculi muscle and a bipolar stimulating electrode (P1 Technologies) adjacent to the left eye. The recording electrodes were for measurement of electromyographic (EMG) activity during blink responses and the bipolar electrode was for shock stimulation. Mice were allowed to recover for 72 h postsurgery.

##### TECC procedure

Mice were placed in a modified operant box (Med Associates) containing a house light and a fan (55 dB), which was housed inside a sound-attenuating box (Med Associates) fitted with acoustic foam. The EMG wires from the animals were plugged into a commutator (P1 Technologies), which enabled uninterrupted electrical signaling while they moved freely. The operant boxes consisted of cabling that connected to a Windows PC equipped with proprietary eyeblink conditioning software (JSA Designs) that recorded EMG activity and delivered the training stimuli [conditioned (CS) and unconditioned (US)]. During each trial, a tone of 80 dB, 2.8 kHz (CS) was presented first and remained on by itself for 380 ms. The tone then terminated and after a 500 ms delay, the shock (US) was delivered and remained on for 100 ms. This 500 ms time window represented the trace interval in which the animal is required to bridge the association between the offset of the tone (CS) and the onset of the shock (US). A total of 90 CS-US trials were presented each session. On every 10th trial, the tone (CS) was presented by itself to test for learning of the conditioned response (CR). In total, there were 100 trials per session with an average intertrial interval of 30 s (18–42 s). Acquisition occurred over six consecutive days (one session per day). Each training session lasted ∼54 min.

##### Data collection

Data were prescreened for “acceptable” and “unacceptable” trials within each session using established criteria in rodent ECC (Skelton, 1988; Stanton and Goodlett, 1998). All relevant measures (below) associated with “acceptable” trials were averaged within session. This was conducted with assistance from proprietary data analysis software (JSA Designs), which divided each trial epoch into four discrete EMG sampling periods: (1) a 280 ms pre-CS baseline before CS onset, (2) a startle response (SR) period during the first 80 ms after CS onset (EMG activity related exclusively to a nonassociative reaction), (3) a 200 ms adaptive CR period that allowed for measuring well-timed CRs before US onset, and (4) a UR (unconditioned response) period which measured EMG activity that occurred from the onset of the US to the end of the trial (140 ms). Any EMG activity that exceeded the pre-CS baseline mean by at least 0.4V (2 standard deviations) was registered by the software as an SR, CR, and/or UR during their respective sampling periods. The reason for analyzing the adaptive CR is that it represents a well-timed eyeblink response just before US onset. Percentage and amplitude of SRs, adaptive CRs, and URs were measured and analyzed as previously described (Tran et al., 2005, 2007; Brown et al., 2007; Rufer et al., 2012).

### Immunofluorescence staining

Adult mice (four to seven months old of both sexes) were deeply anesthetized with Isoflurane until lack of response to toe pinch was recorded, then intracardially perfused with a PBS followed by 10% neutral buffered formalin (fixative). Brains were collected, stored in fixative for 24 h, then incubated in 30% sucrose at 4°C for at least 24 h before sectioning. Coronal sections were cut at 20-μm thickness using a freezing microtome (Leica VT1000 S). Brain sections were washed in PBS, then blocked in 3% normal donkey serum in PBS with 0.03% Triton X-100 (PBST) for 1 h at room temperature. Brain sections were then incubated overnight at room temperature in blocking solution containing anti-NeuN primary antiserum (ABN78, rabbit polyclonal, Millipore; diluted 1:1000). Sections were then washed in PBS and incubated in blocking solution containing anti-rabbit Alexa 488-conjugated secondary antibodies (Life Technologies; diluted 1:500) at room temperature for 1 h. Sections were rinsed in PBS and mounted on Superfrost Plus slides (ThermoFisher) using ProLong Gold with DAPI mounting medium (Invitrogen). Images were taken on a BZ-X800 digital microscope (Keyence). A 10× objective was used to image a whole glass slide containing coronal sections from both genotypes. The stitching function on the BZ-X800 Viewer software (Keyence) was then used to generate images of the whole coronal section.

### SDS-PAGE and immunoblotting

Brain tissue was extracted with NP-40 buffer (Invitrogen) supplemented with inhibitors for proteases and phosphatases (Roche) and 1% deoxycholic acid (DOC). Lysates were then centrifuged at 15,000 × *g* for 15 min at 4°C and the supernatants were used for further analysis. Samples were prepared for standard SDS-PAGE and separated on AnyKD acrylamide gels (Mini-PROTEAN TGX precast gels; Bio-Rad), then transferred onto 0.2-μm pore size PVDF membranes (Millipore) using semi-dry immunoblotting (transfer buffer containing 25 mm Tris, 200 mm glycine and 20% methanol). Membranes were blocked with 5% nonfat milk in TBS-T (tris-buffered saline with 0.1% Tween 20) for 1 h at room temperature, then incubated overnight at 4°C with primary antibodies diluted in 5% BSA in TBS-T. We used the following commercially available primary antibodies: rabbit anti-Rab10 (1:500; Cell Signaling Technology), rabbit anti-GAPDH (1:2000; Sigma), and rabbit anti-GRIN2D (1:500; LSBio). Membranes were washed three times for 15 min in TBS-T, followed by incubation for 2 h at room temperature with HRP-conjugated goat anti-rabbit or goat anti-mouse secondary antibodies (Bio-Rad), diluted 1:5000 in 5% nonfat milk in TBS-T. Membranes were washed three times for 15 min in TBS-T, then incubated with Pierce ECL Plus Western blotting substrate or Pierce ECL Western blotting substrate (for GAPDH) to detect Western blotted proteins using a Bio-Rad Chemidoc touch imaging system. Fiji software was used for Western blot quantification (Schindelin et al., 2012).

### RNA extraction and analysis

RNA was isolated from frozen mouse forebrain tissue. Isolated RNA was purified using the QIAGEN RNeasy kit following the manufacturer’s instructions. The concentration of RNA was determined using Nanodrop (Thermo Fisher Scientific) and the quality of RNA was confirmed using the ECU Genomic Core’s Bioanalyzer system with RNA pico chips.

### Gene expression analysis

Gene expression analysis was performed using NanoString Mouse Neuropathology and Neuroinflammation gene expression panel (NanoString Technologies Inc.). Briefly, RNA isolated from fresh-frozen forebrain tissue was used for experiments; 100 ng of total RNA was hybridized with reporter and capture probes for nCounter Gene Expression code sets (Neuropathology and Neuroinflammation) according to the instructions from manufacturer (NanoString Technologies Inc.). Using the NanoString nSolver Analysis system, data were normalized to spiked positive controls and housekeeping genes. Transcript counts less than the mean of the negative control transcripts plus 2STDEV for each sample were considered background. RNA isolated from five mice per genotype was used for Nanostring mRNA analysis.

### Quantitative RT-PCR (qRT-PCR) analysis

Complementary DNA (cDNA) was synthesized with the High-Capacity cDNA Reverse Transcription kit (Applied Biosystems) following manufacturer’s instructions. Quantitative RT-PCR was performed (Power SYBR Green PCR Master Mix; Applied Biosystems) in triplicate using the Applied Biosystems ViiA 7 system. mRNA gene expression was normalized to mouse 18s ribosomal RNA, with expression fold change calculated using the 2^−ΔΔCt^ method.

The following primer sequences were used for qPCR analysis: Vegfa: forward, CTGAAGGTCAAAGGGAATGTG and reverse, GGACAGAGTCTTGATGATCTC; Prkaa 2: forward, CGGCGCCTTTCCTTGAATAT and reverse, GGCCTGTTCCTCACGGTATTA; Grin2d: forward, CAGCTGCAGGTCATTTTTGA and reverse, GGATCTGCGCACTGACACTA; Syt4: forward, ATGGCTCCTATCACCACCAG and reverse, AGCAGATCCAGGCAAAGAGA; EmCN: forward, AATACCAGGCATCGTGTCAGT and reverse, CTGATTCTCAGTCTTGTTCTGGG; Mouse 18s rRNA: forward, GTAACCCGTTGAACCCCATT and reverse, CCATCCAATCGGTAGTAGCG.

### Experimental design and statistical analysis

For all experiments mice of both genotypes were processed in parallel. Both sexes were used for adult behavior studies, immunofluorescence staining, biochemistry, and gene expression studies. GraphPad Prism (version 9.4.1 for Windows, GraphPad Software) was used for the majority of statistical analysis. Student’s *t* test was used to compare two independent datasets. For multiple comparisons, we used ANOVA followed by Dunnett’s test. Differences between genotypes or samples were considered significant at *p* < 0.05. This software was also used to calculate a 95% confidence interval (CI) for a difference between means where applicable (Calin-Jageman and Cumming, 2019; Ho et al., 2019; Bernard, 2021). In figures and tables, data are reported as mean ± SEM, unless otherwise stated.

## Results

### Normal gross brain morphology and physical attributes of Rab10^+/−^ mice

**Figure 1.**
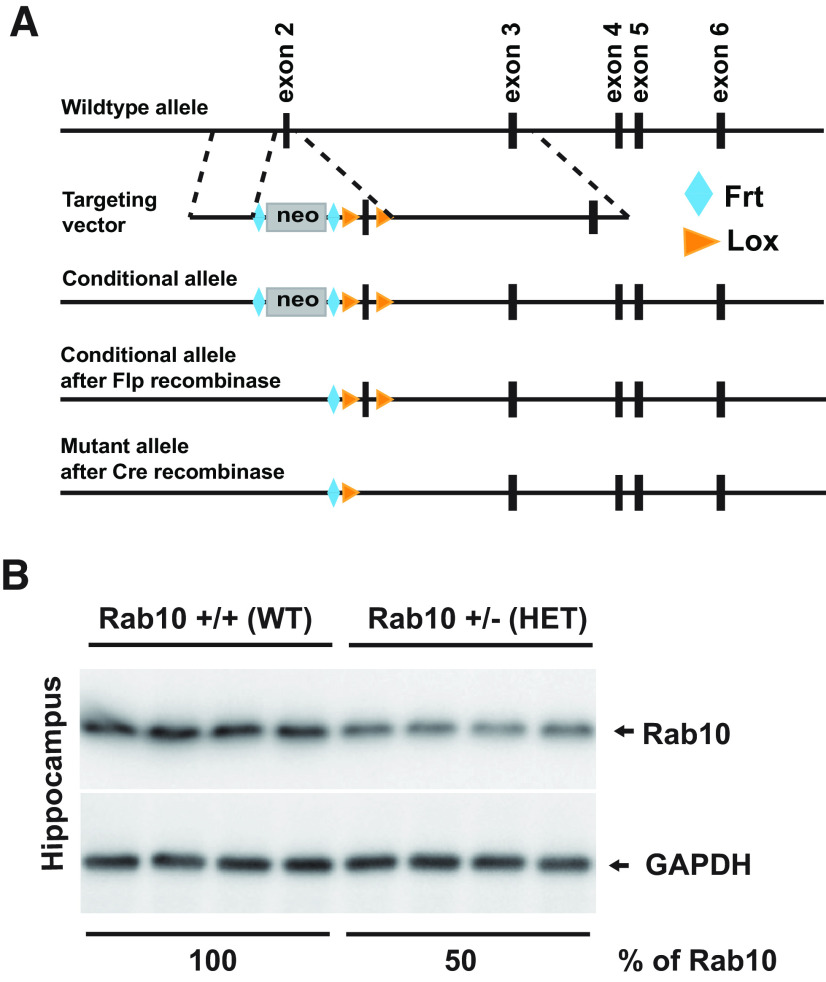
Generation of Rab10 conditional and constitutive knock-out (KO) mice. ***A***, Schematic drawing of the targeting strategy used to generate Rab10 conditional and constitutive knock-out (KO) mouse lines. In Rab10 conditional KO line, exon 2 of *Rab10* gene was flanked by two lox sites. A frt-flanked neo cassette was placed upstream of the start of exon 2 and further removed by Flp recombination. Rab10 constitutive KO mouse line was generated by crossing Rab10 conditional heterozygous mouse line with CMV-Cre mouse line. Expression and translation of this modified Rab10 locus resulted in a frame shift product of Rab10 protein. ***B***, Immunoblots show that in the hippocampus of Rab10^+/−^ (heterozygous, HET) mice, the level of Rab10 is reduced to 50% compared with the level in the brain of Rab10^+/+^ (wild-type; WT) littermate mice. Numbers under blots represent GAPDH-normalized Rab10 level in four independent experiments.

**Figure 2.**
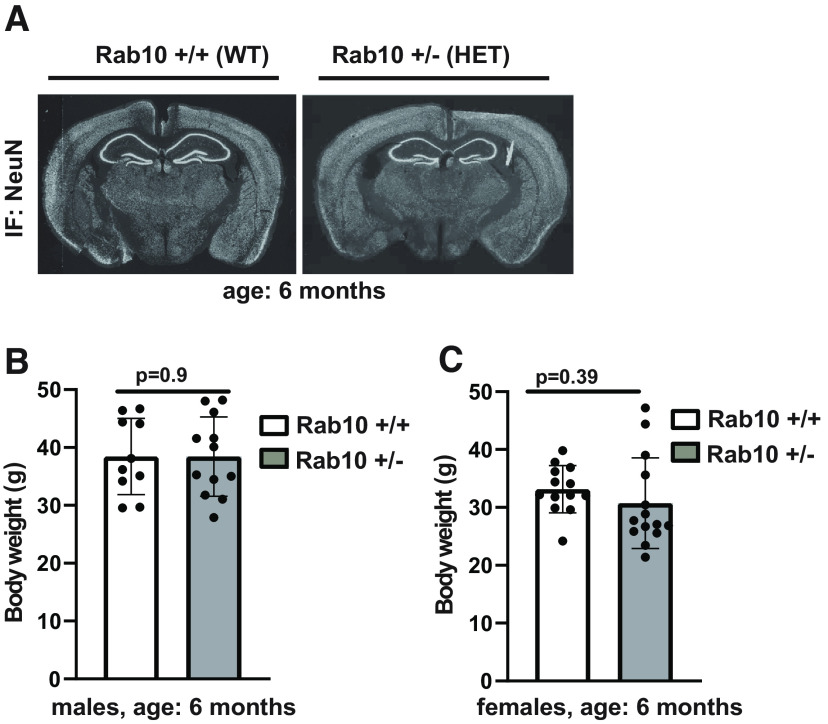
Normal brain gross anatomy and physical attributes of Rab10^+/−^ mice. ***A*,** NeuN immunohistochemistry of coronal sections from six-month-old Rab10^+/−^ and nontransgenic littermate Rab10^+/+^ mice show normal brain morphology in Rab10-deficient mice. Similar results were obtained in three independent experiments. ***B***, ***C***, The body weight (in grams) of Rab10^+/−^ and Rab10^+/+^ mice is not significantly different in males, nor in females. ***B***, The number of male mice was 10 for Rab10^+/+^ and 12 for Rab10^+/−^ genotype. Error bars indicate SEM. Two-tailed unpaired *t* test, *p* = 0.9. ***C***, The number of female mice was 13 for Rab10^+/+^ and 14 for Rab10^+/−^ genotype. Error bars indicate SEM. Two-tailed unpaired *t* test, *p* = 0.39.

Rab10 conditional knock-out mice were generated from C57BL/6 mice at Duke University Transgenic Core Facility using a strategy described in Materials and Methods (Fig. 1*A*). To generate Rab10 constitutive knock-out mice, we further crossed Rab10 cKO mice with CMV-Cre mice, which express Cre in all tissues during early embryogenesis. Because of the previously reported embryonic lethality caused by rab10 deletion (Lv et al., 2015), we were able to generate heterozygous knock-out mice for Rab10 (Rab10^+/−^) instead of homozygous. Immunoblotting analysis showed an ∼50% reduction in the expression of Rab10 in the hippocampus of Rab10^+/−^ (heterozygous; HET) mice compared with their nontransgenic littermates (Rab10^+/+^; wild-type; WT; Fig. 1*B*). To assess whether Rab10 reduction affects gross brain structure, we compared the overall brain morphology between Rab10^+/+^ and Rab10^+/−^ mice. NeuN immunostaining of coronal sections was indistinguishable between the two genotypes (Fig. 2*A*). Considering Rab10 is involved in metabolic regulation (Brumfield et al., 2021), we compared the body weight between Rab10^+/−^ mice and their nontransgenic littermates (Rab10^+/+^). The body weight (in grams) of Rab10^+/−^ and Rab10^+/+^ mice (Fig. 2*B*,*C*) was indistinguishable for both males (Rab10^+/+^: 38.45 ± 12.15, *n* = 10; Rab10^+/−^: 38.71 ± 11.09, *n* = 12; *t*_(9)_ = 0.251, *p* = 0.9, 95% CI [−6.457, 5.169]; SEM; two-tailed unpaired *t* test) and females (Rab10^+/+^: 33.15 ± 9.19, *n* = 13; Rab10^+/−^: 30.71 ± 8.2; *n* = 14; *t*_(12)_ = 0.728, *p* = 0.39, 95% CI [−6.898, 3.502]; SEM; two-tailed unpaired *t* test).

### Differentially expressed genes between Rab10^+/−^ and Rab10^+/+^ mice

**Figure 3.**
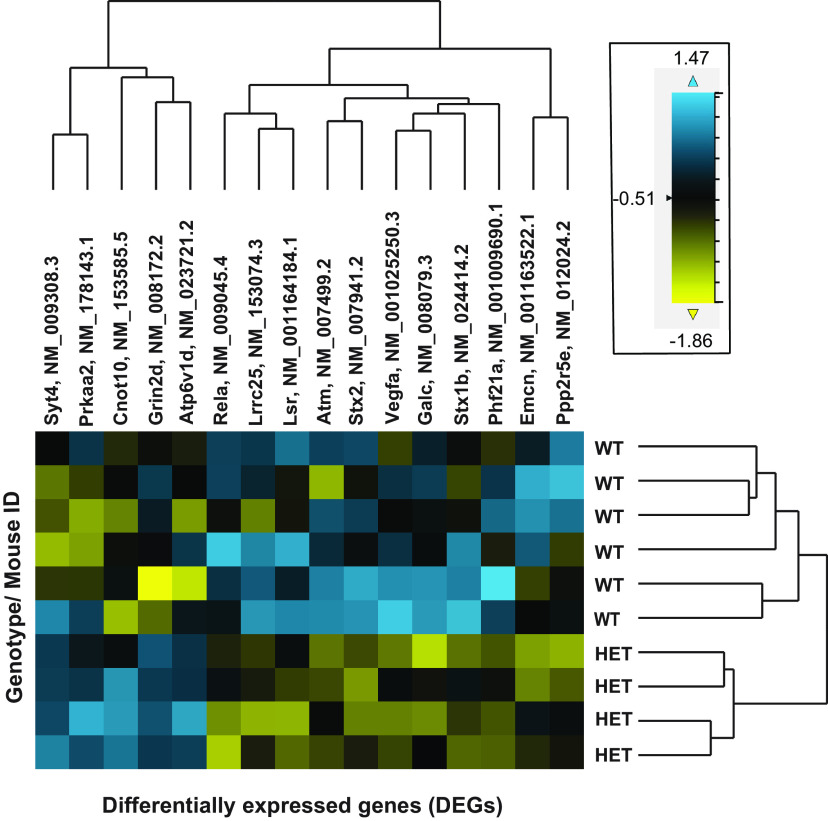
NanoString nCounter profiling identified several differentially expressed genes in the brain of Rab10^+/−^ mice. Heatmap depicts the hierarchical clustering of 16 differentially expressed genes (DEGs) between genotypes, of which 11 were significantly downregulated (Stx2, Lsr, Lrrc25, Vegfa, Galc, Phf21a, Rela, Emcn, Atm, Ppp2r5e, Stx1b, yellow; Table 1) and five were significantly upregulated (Cnot10, Atp6v1d, Syt4, Prkaa2, Grin2d, blue; Table 2) in the forebrain of Rab10^+/−^ mice compared with their Rab10^+/+^ littermates.

**Figure 4.**
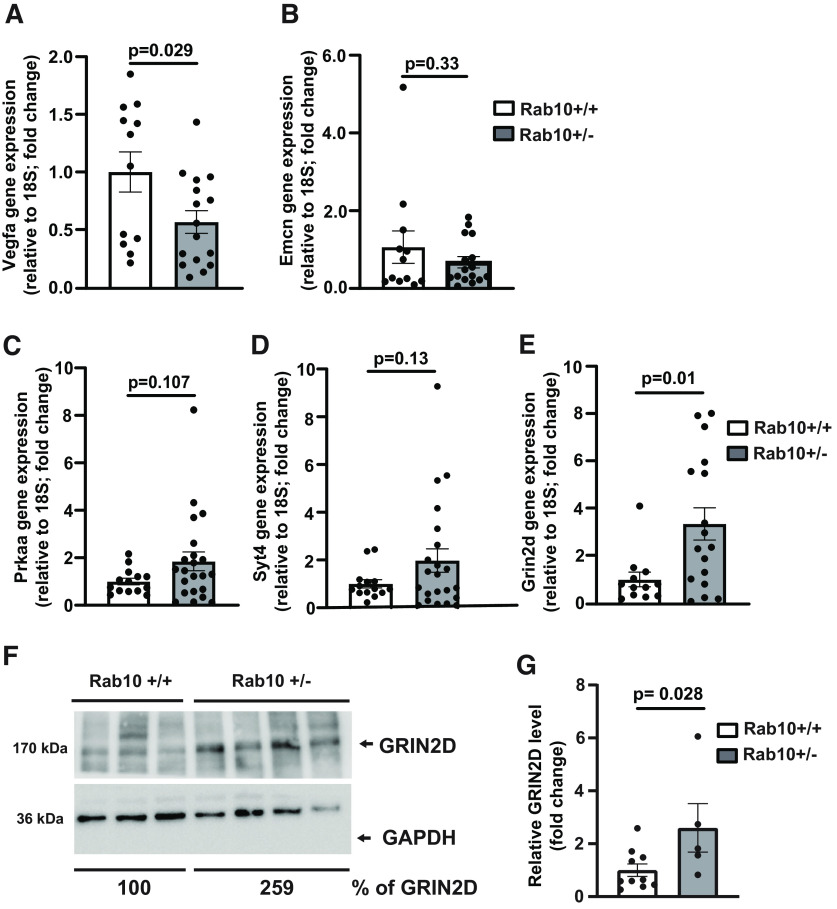
Quantitative RT-PCR and Western blotting to validate a subset of DEGs in the brain of Rab10^+/−^ mice. ***A***, Vegfa gene expression evaluated by qRT-PCR. The number of mice was 12 for Rab10^+/+^ and 16 for Rab10^+/−^ genotype. Error bars indicate SEM. Two-tailed unpaired *t* test, *p* = 0.029. ***B***, Emcn gene expression evaluated by qRT-PCR. The number of mice was 12 for Rab10^+/+^ and 16 for Rab10^+/−^ genotype. Error bars indicate SEM. Two-tailed unpaired *t* test, *p* = 0.33. ***C***, Prkaa gene expression evaluated by qRT-PCR. The number of mice was 14 for Rab10^+/+^ and 22 for Rab10^+/−^ genotype. Error bars indicate SEM. Two-tailed unpaired *t* test, *p* = 0.107. ***D***, Syt4 gene expression evaluated by qRT-PCR. The number of mice was 14 for Rab10^+/+^ and 22 for Rab10^+/−^ genotype. Error bars indicate SEM. Two-tailed unpaired *t* test, *p* = 0.13. ***E***, Grin2d gene expression evaluated by qRT-PCR. The number of mice was 12 for Rab10^+/+^ and 17 for Rab10^+/−^ genotype. Error bars indicate SEM. Two-tailed unpaired *t* test, *p* = 0.013. ***F***, Representative images of GRIN2D protein expression in the hippocampus as evaluated by Western blotting. Numbers under the blots represent relative GRIN2D levels, normalized to the house keeping protein, GAPDH. ***G***, Graph showing relative GRIN2D protein expression in the hippocampus evaluated by Western blotting from 10 Rab10^+/+^ and 5 Rab10^+/−^ mice. Error bars indicate SEM. Two-tailed unpaired *t* test, *p* = 0.028.

To gain insights into the signaling pathways driving Rab10-dependent neuroresilience, we employed the NanoString nCounter platform to interrogate gene expression profiles in our cohorts. We isolated RNA from the frontal cortical tissue of Rab10^+/−^ and Rab10^+/+^ mice, and identified 16 differentially expressed genes (DEGs), of which 11 were significantly downregulated (Table 1) and five of which were significantly upregulated (Table 2). We used a heatmap analysis to depict hierarchical clustering of upregulated (blue) and downregulated (yellow) genes (Fig. 3). Gene expression profiling also revealed distinct pathways and cell-type changes in the Rab10^+/−^ mice compared with their control Rab10^+/+^ littermates (Table 3). The identified DEGs are associated with signaling pathways mediating neuroinflammation (Vegfa, Galc, Lrrc25); neuroplasticity, development, and aging (Vegfa, Phf21a, Rela, Prkaa2, Emcn, Atm); metabolism (Stx2, Lsr, Atp6v0d1, Galc, Rela, Prkaa2, Ppp2r5e); compartmentalization and structural integrity (Stx2, Atp6v0d1, Syt4, Grin2d, Stx1b); neuron-glia interaction (Rela) and neurotransmission (Stx2, Atp6v0d1, Syt4, Rela. Grin2d, Ppp2r5e, Stx1b). We validated several significantly altered genes by quantitative RT-PCR (qRT-PCR) analysis (Fig. 4*A****–****E*), which was performed on RNA isolated from the corresponding frozen tissue specimens used for NanoString nCounter analysis (Prokop et al., 2019; Crist et al., 2021). Similar changes were observed in the subset of genes tested (Vegfa, Fig. 4*A*; Rab10^+/+^: 1.0 ± 0.28, *n* = 12; Rab10^+/−^: 0.56 ± 0.14, *n* = 16; *t*_(26)_ = 2.304, *p* = 0.029, 95% CI [−1.34, −0.077]; SEM; Emcn, Fig. 4*B*; Rab10^+/+^: 1.0 ± 0.30, *n* = 12; Rab10^+/−^: 0.67 ± 0.16, *n* = 16; *t*_(27)_ = 0.827, *p* = 0.33, 95% CI [−0.762, 0.922]; SEM; Prkaa, Fig. 4*C*; Rab10^+/+^: 1.0 ± 0.27, *n* = 14; Rab10^+/−^: 1.84 ± 039, *n* = 22; *t*_(34)_ = 1.654, *p* = 0.107, 95% CI [−0.206, 2.008]; SEM; Syt4, Fig. 4*D*; Rab10^+/+^: 1.0 ± 0.27, *n* = 14; Rab10^+/−^: 2.03 ± 0.43, *n* = 22; *t*_(34)_ = 1.580, *p* = 0.13, 95% CI [−0.339 2.710]; SEM; Grin2d, Fig. 4*E*; Rab10^+/+^: 1.0 ± 0.29, *n* = 12; Rab10^+/−^: 3.3 ± 0.82, *n* = 17; *t*_(28)_ = 2.875, *p* = 0.01, 95% CI [0.746, 4.440]; SEM), with two individual genes (Vegfa and Grin2d) showing statistical significance in the qRT-PCR analysis. Next, we validated the NanoString and qRT-PCR results using biochemical analysis of the corresponding frozen tissue specimens. Western blot analysis of GRIN2D protein level in hippocampal samples (Fig. 4*F*) showed increased expression of GRIN2D in Rab10^+/−^ mice (Rab10^+/+^: 1.0 ± 0.31, *n* = 10; Rab10^+/−^: 2.6 ± 1.16, *n* = 5; *t*_(13)_ = 2.249, *p* = 0.028, 95% CI [0.063, 3.132]; SEM; Fig. 4*G*).

**Table 1:** Genes significantly downregulated in the brain of Rab10^+/−^ mice

DEG: downregulated in Rab10^+/−^	Accession #	Rab10^+/+^ average normalized count/probe	Rab10^+/−^ average normalized count/probe	*p*-value	*t*-statistics: Rab10^+/−^ vs Rab10^+/+^	95% CI
Stx2	NM_007941.2	79.68 ± 5.14	68.7 ± 2.44	*p* = 0.001	−4.668	4.65, 17.52
Lsr	NM_001164184.1	24.88 ± 3.14	20.12 ± 1.93	*p* = 0.016	−3.033	0.768, 8.947
Lrrc25	NM_153074.3	10.14 ± 3.77	5.45 ± 1.16	*p* = 0.018	−2.959	0.797, 9.738
Vegfa	NM_001025250.3	184.1 ± 23.35	153.22 ± 11.38	*p* = 0.019	−2.917	2.482, 61.126
Galc	NM_008079.3	116.24 ± 13.5	88.47 ± 12.65	*p* = 0.021	−3.179	8.126, 47.326
Phf21a	NM_001109690.1	30.57 ± 7.23	22.73 ± 1.83	*p* = 0.022	−2.955	0.9501, 16.01
Rela	NM_009045.4	55.87 ± 8.51	40.92 ± 6.95	*p* = 0.023	−3.047	3.200, 26.835
Emcn	NM_001163522.1	33.41 ± 5.43	26.31 ± 3.46	*p* = 0.033	−2.582	0.200, 14.410
Atm	NM_007499.2	55.62 ± 6.74	47.89 ± 2.7	*p* = 0.041	−2.483	0.820
Ppp2r5e	NM_012024.2	281.46 ± 17.47	260.16 ± 10.96	*p* = 0.044	−2.385	0.8508. 42.32
Stx1b	NM_024414.2	2874.87 ± 196.82	2661.65 ± 95.64	*p* = 0.049	−2.328	0.035, 435.2

Table shows differentially expressed genes (DEGs) that are significantly downregulated in the forebrain of Rab10^+/−^ mice. The number of mice was 6 for Rab10^+/+^ and 4 for Rab10^+/−^ genotype. Data are means± SEM; two-tailed unpaired *t* test was performed and *p* < 0.05 was used for initial filtering and DEG selection.

**Table 2:** Genes significantly upregulated in the Rab10^+/−^ brain

DEG: upregulated in Rab10^+/−^	Accession #	Rab10^+/+^ average normalized count/probe	Rab10^+/−^ average normalized count/probe	*p*-value	*t* statistics: Rab10^+/−^ vs Rab10^+/+^	95% CI
Cnot10	NM_153585.5	176.14 ± 8.9	199.06 ± 10.04	*p* = 0.008	3.689	9.032, 36.835
Atp6v1d	NM_023721.2	1610.64 ± 76.48	1721.72 ± 39.5	*p* = 0.019	2.951	−12.974, −206.808
Syt4	NM_009308.3	902.25 ± 44.03	962.42 ± 12.4	*p* = 0.019	3.146	6.351, 112.379
Prkaa2	NM_178143.1	139.58 ± 11.37	155.96 ± 7.9	*p* = 0.027	2.694	9.032, 36.835
Grin2d	NM_008172.2	15.26 ± 4.81	22.48 ± 0.99	*p* = 0.039	2.729	−0.887, −12.210

Table shows differentially expressed genes that are significantly upregulated in the forebrain of Rab10^+/−^ mice. The number of mice was 6 for Rab10^+/+^ and 4 for Rab10^+/−^ genotype. Data are mean ± SEM; two-tailed unpaired *t* test was performed and *p* < 0.05 was used for initial filtering and DEG selection.

**Table 3:** Pathway and cell-type changes in the brain of Rab10^+/−^ mice

DEG	Neuroinflammation	Neuroplasticity, development, and aging	Metabolism	Compartmentalization and structural integrity	Neuron–glia interaction	Neurotransmission
Stx2 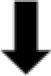	−	−	+	+	−	+
Lsr 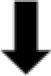	−	−	+	−	−	−
Lrrc25 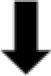	+	−	−	−	−	−
Vegfa 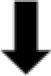	+	+	−	−	−	−
Atp6v0d1 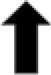	−	−	+	+	−	+
Syt4 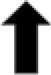	−	−	−	+	−	+
Galc 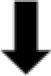	+	−	+	−	−	−
Phf21a 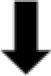	−	+	−	−	−	−
Rela 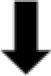	−	+	+	−	+	+
Prkaa2 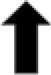	−	+	+	−	−	−
Emcn 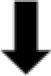	−	+	−	−	−	−
Grin2d 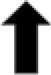	−	−	−	+	−	+
Atm 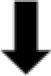	−	+	−	−	−	−
Ppp2r5e 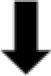	−	−	+	−	−	+
Stx1b 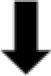	−	−	−	+	−	+

Table shows enriched pathways and cell-type interactions in the Rab10^+/−^ brain. The number of mice was 6 for Rab10^+/+^ and 4 for Rab10^+/−^ genotype. Up and down arrows indicate upregulated and downregulated genes, respectively. +: DEG is associated with a pathway/cell-type interaction; −: DEG is not associated with a pathway/cell-type interaction.

### Behavioral characterizations of Rab10^+/−^ mice

**Figure 5.**
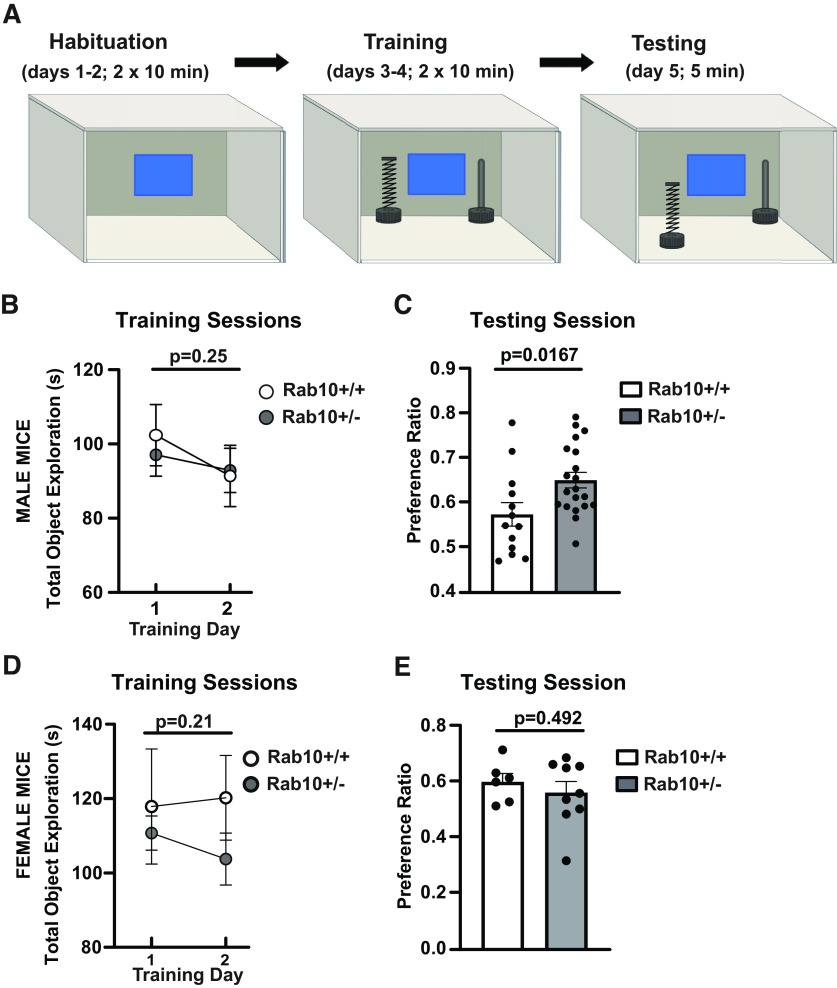
Improved performance of Rab10^+/−^ mice in object in place spatial memory task is sex dependent. ***A***, Diagram of object-in-place (OIP) memory test. Created with BioRender. ***B***, Graph shows no significant difference between genotypes in averaged duration of object exploration during training. The number of male mice was 13 for Rab10^+/+^ and 20 for Rab10^+/−^ genotype. Error bars indicate SEM. Two-tailed unpaired *t* test, *p* = 0.25. ***C***, Male Rab10^+/−^ mice exhibit significant discrimination of the familiar and novel object locations and exhibit strong preference for the object in the novel location as compared with their Rab10^+/+^ littermates during OIP testing session. Error bars indicate SEM. Two-tailed unpaired *t* test, *p* = 0.0167. ***D***, Graph shows no significant difference between genotypes in averaged duration of object exploration during training. The number of female mice was six for Rab10^+/+^ and nine for Rab10^+/−^ genotype. Error bars indicate SEM. Two-tailed unpaired *t* test, *p* = 0.21. ***E***, Female Rab10^+/−^ mice do not exhibit a stronger preference for the object in the novel location as compared with their Rab10^+/+^ littermates during OIP testing session. Error bars indicate SEM. Two-tailed unpaired *t* test, *p* = 0.492.

We examined the behavior of Rab10^+/−^ mice and their control littermates on several standard tasks. Baseline behaviors (Table 4) showed no statistically significant difference between genotypes as indicated by similar levels of anxiety-like behavior in the open field and elevated plus maze test. However, during the OF test, there was a statistically significant difference between genotypes in total distance moved (Rab10^+/+^: 5289.2 ± 300.1, *n* = 16; Rab10^+/−^: 6102.1 ± 260.6, *n* = 14; *t*_(28)_ = 2.056, *p* = 0.05, 95% CI = [3.01, 1623]; SEM). This is likely a result of the Rab10^+/−^ mice having increased interest in contextual novelty. The fact that both groups performed well indicates that it is not likely that this difference is because of a decrease in learning ability. In addition, working memory measured by spontaneous alteration test was intact in Rab10^+/−^ mice. Therefore, reducing Rab10 expression had no statistically significant effect on motor activity, anxiety-like behavior, and working memory in mice. It should be noted, that if tested in other behavioral tasks, these mice may demonstrate performance deficits. Next, we performed the Morris water maze (MWM) task, a commonly used test for hippocampal-dependent spatial learning and memory. We evaluated swim speed and latency to a platform in the visible platform version of the MWM, and found no statistically significant difference between genotypes, indicating no impairment of escape motivation, vision, and motor skills. Subsequently, the mice were trained in the hidden platform version of MWM using four trials per day for 8 d, with an intertrial interval of 20–30 min. On day 9, we administered a probe test without the platform present. There was no statistically significant difference between genotypes in the MWM test (Table 4). Next, we conducted hippocampal-dependent trace fear conditioning in which the conditioned stimulus (CS) and unconditioned stimulus (US) become associated across a stimulus-free time interval (the “trace”). Both genotypes exhibited greater freezing during the “anticipatory” trace period as compared with the tone, suggesting successful hippocampal-dependent learning. We did not find significant differences between genotypes (Table 4). We also evaluated spatial and contextual memory performance in an object-in-place memory task (OIP). Memory in the OIP task depends on the interaction between the hippocampus, perirhinal cortex and medial prefrontal cortex (Barker and Warburton, 2015). Briefly, we trained mice to learn the location of two objects, in a familiar arena. During a subsequent test session, one object was moved to a new location in the arena. If the mouse recognized the location change, it would exhibit a preference for exploring the moved object. The experimental design of OIP test is presented in Figure 5*A*. Mice were habituated to the arena for 10 min/d for two consecutive days. The habituation sessions were followed by 2 d of training that consisted of 10 min/d in the arena that now contained two toy objects. For male mice (Fig. 5*B*,*C*), during the OIP training sessions there was no difference in total object exploration time (s) between genotypes (mean total object exploration for the first training session: Rab10^+/+^ = 102.36 ± 8.23; Rab10^+/−^ = 97.088 ± 5.74; *t*_(13)_ = 0.5235, *p* = 0.56, 95% CI [−36.69, 22.37]; mean total object exploration for second training session: Rab10^+/+^ = 91.39 ± 8.27; Rab10^+/−^ = 92.86 ± 5.99; *t*_(12)_ = 1.145, *p* = 0.25, 95% CI [−44.30, 13.78]; *n* = 13 for Rab10^+/+^ and *n* = 20 for Rab10^+/−^; Fig. 5*B*). One day after the completion of the training session, we performed a 5 min OIP test session in the familiar arena, with one toy object transferred to the opposing southern corner. We found that male Rab10^+/−^ mice preferred the object in the novel location significantly more than their control littermates (preference ratio: Rab10^+/+^ = 0.57 ± 0.03; Rab10^+/−^ = 0.65 ± 0.02; *t*_(31)_ = 2.530, *p* = 0.0167, 95% CI [0.015, 0.139]; Fig. 5*C*). Next, we performed the OIP experiments using age-matched female Rab10^+/−^ mice and their control littermates (Fig. 5*D*,*E*). Similar to male mice, during the OIP training session there was no difference in total object exploration time(s) between genotypes (mean total object exploration for the first training session: Rab10^+/+^ = 117.87 ± 15.5; Rab10^+/−^ = 110.71 ± 4.63; *t*_(13)_ = 0.5235, *p* = 0.56, *p* = 0.61, 95% CI [−36.69, 22.37]; mean total object exploration for second training session: Rab10^+/+^ = 120.22 ± 11.4; Rab10^+/−^ = 103.75 ± 6.98; *t*_(13)_ = 1.145, *p* = 0.21, [−44.30, 13.78]; Fig. 5*D*). However, during the object location preference testing session, the females did not reveal significant difference between genotypes (preference ratio: Rab10^+/+^ = 0.59 ± 0.03; Rab10^+/−^ = 0.55 ± 0.04; *t*_(13)_ = 0.708, *p* = 0.492, 95% CI [−0.039, 0.079]; *n* = 6 for Rab10^+/+^ and *n* = 9 for Rab10^+/−^; Fig. 5*E*). These results suggest that there may be a sex-dependent effect of Rab10 signaling on spatial recognition memory performance.

**Table 4:** Baseline behaviors in male Rab10^+/+^ and Rab10^+/−^ mice

Behavioral test	Measurement	Testing for	Rab10^+/+^ (*n* = 16)	Rab10^+/−^ (*n* = 14)	Statistical significance	Statistical test
Open field	Distance moved (cm)	Activity and motor performance	5289.2 ± 300.1	6102.1 ± 260.6	*p* = 0.05 (significant)	*t* test
Open field	Exit latency (s)	Anxiety	4.54 ± 0.17	5.08 ± 0.26	*p* = 0.11 (not significant)	*t* test
Elevated plus maze	Time spent in open arm (%)	Anxiety-like behavior	45.11 ± 11.8	46.26 ± 15.0	*p* = 0.97 (not significant)	*t* test
Spontaneous alternation	Alternation success between two mazes (%)	Working memory	85.71 ± 9.71	75.00 ± 11.18	*p* = 0.48 (not significant)	*t* test
MWM-VPT	Latency to visible platform (s)	Visual acuity	13.24 ± 1.68	13.36 ± 1.05	*p* = 0.91 (not significant)	*t* test
MWM-acquisition	Latency to platform during final training session (s)	Navigational learning	20.12 ± 2.56	19.85 ± 3.85	*p* = 0.88 (not significant)	*t* test
MWM–probe test	Distance to platform center during probe 3 (cm)	Hippocampal-dependent navigation	39.05 ± 2.72	42.41 ± 3.01	*p* = 0.34 (not significant)	*t* test
Trace fear conditioning	Freezing during trace of tone test (%)	Hippocampal functioning	67.98 ± 3.45	71.05 ± 2.66	*p* = 0.81 (not significant)	*t* test

This table shows the behavioral tests to identify potential abnormalities in baseline behaviors of Rab10^+/−^ mice. The number of mice was 16 for Rab10^+/+^ and 14 for Rab10^+/−^ genotype. With the exception of open field test (distance moved), on all measurements, there was no statistically significant difference between Rab10^+/−^ mice and their Rab10^+/+^ littermates.

**Figure 6.**
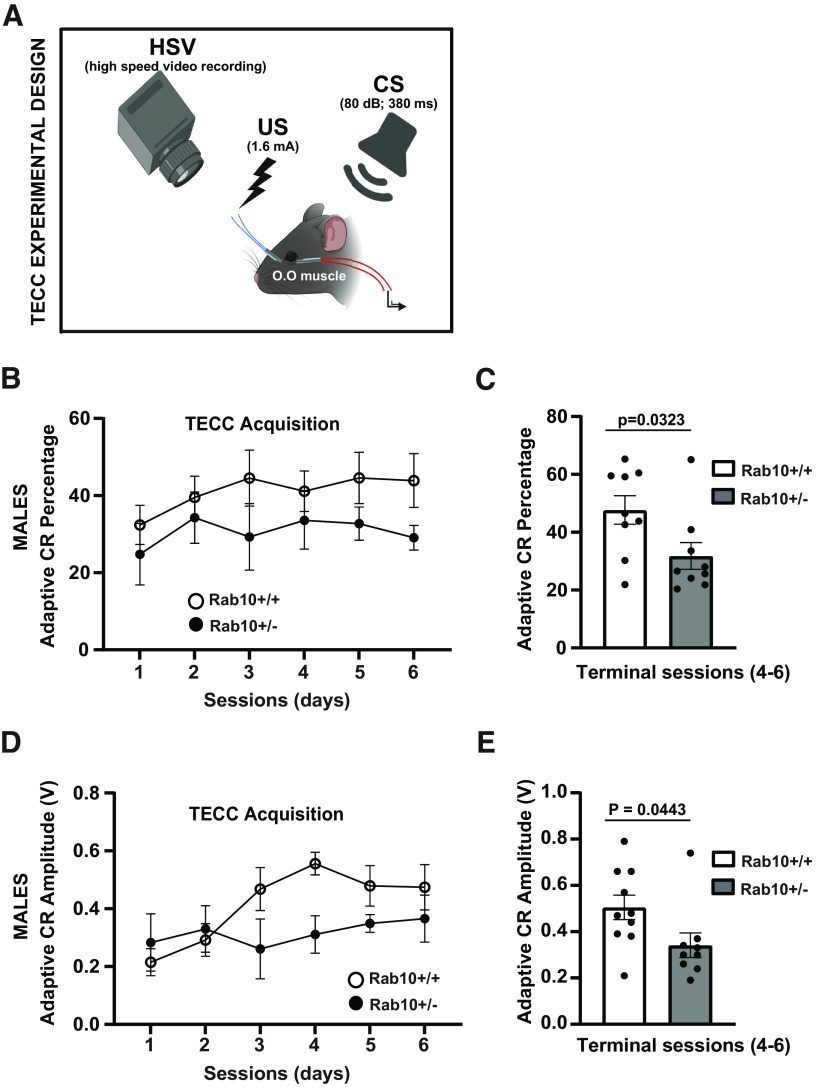
Impaired performance of male Rab10^+/−^ mice in trace eyeblink classical conditioning (TECC). ***A***, Experimental design of TECC: HSV, high speed video recording; CS, conditioned stimulus (tone; 80 dB for 380 ms); US, unconditioned stimulus (a mild shock to the eyelid; 1.6 mA); O.O muscle (orbicularis oculi muscle); created with BioRender. ***B***, Acquisition curve of adaptive conditioned response (CR) percentage. The number of mice was 10 for Rab10^+/+^ and 9 for Rab10^+/−^ genotype. ***C***, Graph indicates that Rab10^+/−^ male mice exhibit a statistically significant deficit in the adaptive conditioned response percentage during the terminal sessions (4–6) of the TECC acquisition curve. Error bars indicate SEM. Two-tailed unpaired *t* test, *p* = 0.0323. ***D***, Acquisition curve of adaptive conditioned response (CR) amplitude. The number of mice was 10 for Rab10^+/+^ and 9 for Rab10^+/−^ genotype. ***E***, Graph shows that Rab10^+/−^ mice exhibit a statistically significant deficit in the adaptive CR amplitude during the terminal sessions (4–6) of the TECC acquisition curve. Error bars indicate SEM. Two-tailed unpaired *t* test, *p* = 0.0443.

**Figure 7.**
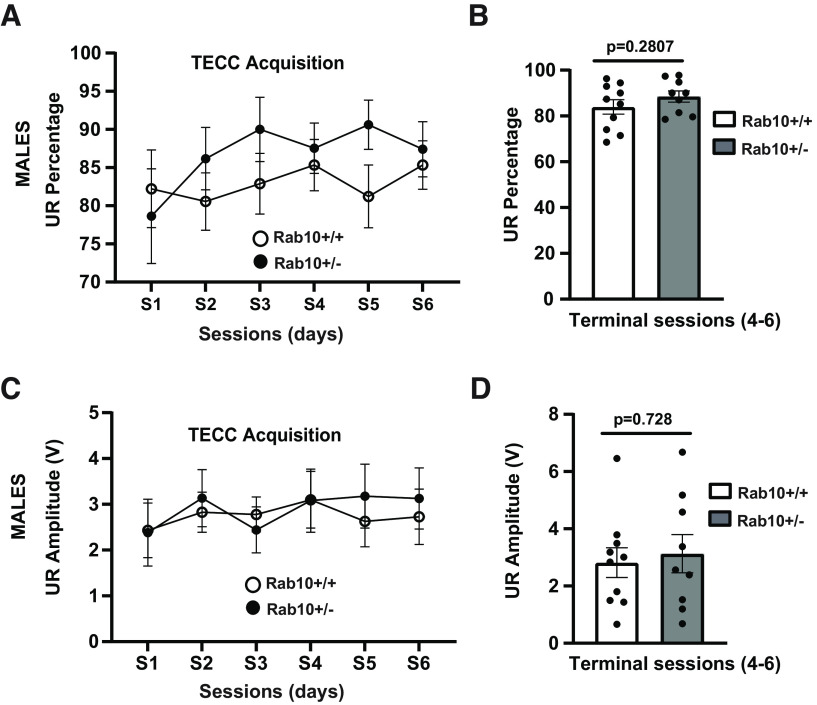
Male Rab10^+/−^ mice exhibit normal sensorimotor performance in TECC paradigm. ***A***, Acquisition curve of unconditioned response (UR) percentage. The number of mice was 10 for Rab10^+/+^ and 9 for Rab10^+/−^ genotype. ***B***, Graph shows that there is no significant difference between genotypes in UR percentage during the terminal sessions (4–6) of the TECC acquisition curve. Error bars indicate SEM. Two-tailed unpaired *t* test, *p* = 0.2807. ***C***, Acquisition curve of UR amplitude. The number of mice was 10 for Rab10^+/+^ and 9 for Rab10^+/−^ genotype. ***D***, Graph indicates that Rab10^+/−^ mice do not exhibit deficit in UR amplitude during the terminal sessions (4–6) of the TECC acquisition curve. Error bars indicate SEM. Two-tailed unpaired *t* test, *p* = 0.728.

It has been hypothesized that cue-response association (such as eyeblink conditioning) occurs in the primary motor cortex (M1) and requires the activity of NMDA receptors (Hasan et al., 2013). Eyeblink classical conditioning (ECC) is widely used to understand the mechanisms of learning and memory consolidation. In ECC, a CS (tone) is paired with the US (a mild shock to the eyelid). In untrained animals, the US applied alone will elicit an unconditioned response (UR, eyeblink). Following repetitive CS-UR pairing, the CS alone will elicit an eyeblink as a conditioned response (CR). In trace eyeblink classical conditioning (TECC), there is a stimulus-free period (trace interval) between CS and US. The TECC requires the participation of the cerebellum, cortex, and hippocampus (Tran et al., 2007, 2017). To evaluate the involvement of Rab10 in cue-response association, we performed TECC in both male and female mice. The experimental design of TECC test is presented in Figure 6*A*. Acquisition of trace conditioned responses indicated that male Rab10^+/−^ mice exhibit a statistically significant deficit in acquisition of CR during the terminal sessions (4–6) of the acquisition curve (Fig. 6*B–E*). The difference between genotypes was significant both in adaptive CR percentage (Rab10^+/+^= 44.261 ± 13.996; Rab10^+/−^ = 31.793 ± 10.597; *t*_(41)_ = 3.335, *p* = 0.0323, 95% CI [6.268, 25.510]; Fig. 6*B*,*C*) and adaptive CR amplitude (Rab10^+/+^= 0.505 ± 0.159; Rab10^+/−^ = 0.341 ± 0.113; *t*_(41)_ = 2.883, *p* = 0.0443, 95% CI [0.056, 0.317]; Fig. 6*D*,*E*). To verify whether learning was affected by sensorimotor performance, we evaluated unconditioned responses. The two genotypes showed no significant difference for both UR percentage (Rab10^+/+^ = 83.963 ± 26.551; Rab10^+/−^ = 88.518 ± 29.506; *t*_(41)_ = 0.489, *p* = 0.2807, 95% CI [−4.902, 8.032]; Fig. 7*A*,*B*) and UR amplitude (Rab10^+/+^ = 2.817 ± 0.89; Rab10^+/−^ = 3.130 ± 1.043; *t*_(41)_ = 0.200, *p* = 0.728, 95% CI [−1.216, 0.997]; Fig. 7*C*,*D*).

**Figure 8.**
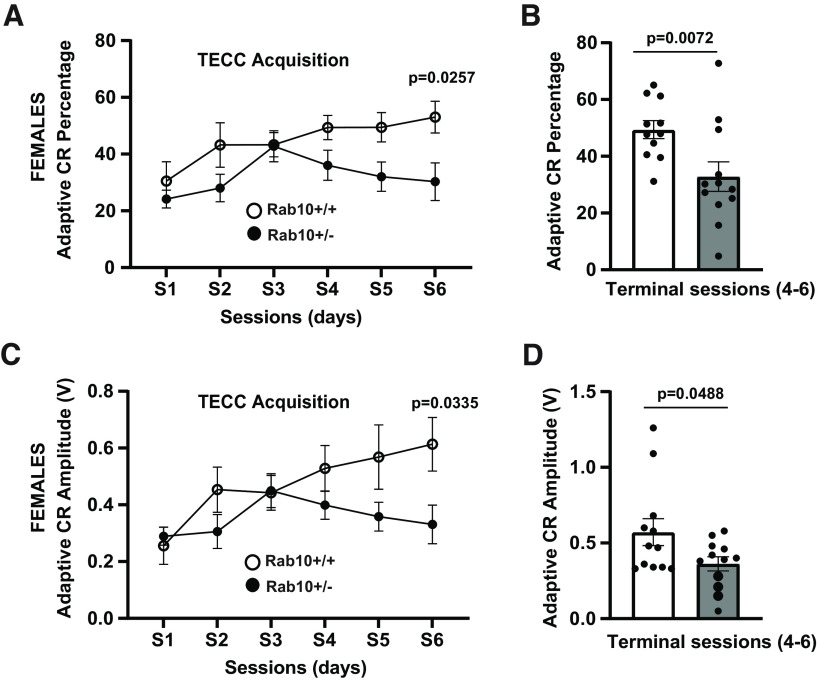
Impaired performance of female Rab10^+/−^ mice in TECC. ***A***, Acquisition curve of adaptive conditioned response (CR) percentage. The number of mice was 11 for Rab10^+/+^ and 12 for Rab10^+/−^ genotype. Female Rab10^+/−^ mice exhibit a statistically significant deficit in the CR percentage during the terminal S6 session of the TECC acquisition curve. Two-tailed unpaired *t* test, *p* = 0.0257. ***B***, Graph indicates that female Rab10^+/−^ mice exhibit a significant deficit in the adaptive conditioned response percentage during the terminal sessions (4–6) of the TECC acquisition curve. Error bars indicate SEM. Two-tailed unpaired *t* test, *p* = 0.0072. ***C***, Acquisition curve of adaptive CR amplitude. The number of mice was 11 for Rab10^+/+^ and 12 for Rab10^+/−^ genotype. Female Rab10^+/−^ mice exhibit a statistically significant deficit in the CR amplitude during the terminal S6 session of the TECC acquisition curve. Two-tailed unpaired *t* test, *p* = 0.0335. ***D***, Graph shows that female Rab10^+/−^ mice exhibit a significant deficit in the CR amplitude during the terminal sessions (4–6) of the TECC acquisition curve. Error bars indicate SEM. Two-tailed unpaired *t* test, *p* = 0.0488.

Next, we analyzed the acquisition of trace CR in female mice (Fig. 8). Our data show that female Rab10^+/−^ mice have a statistically significant deficit both in adaptive CR percentage (Rab10^+/+^= 53.038 ± 15.310; Rab10^+/−^ = 30.283 ± 8.742; *F*_(5,127)_ = 2.502, *p* = 0.0257; Fig. 8*A*) and adaptive CR amplitude (Rab10^+/+^= 0.613 ± 0.177; Rab10^+/−^ = 0.330 ± 0.095; *F*_(5,132)_ = 2.419, *p* = 0.0335; Fig. 8*C*) in the last session (S6) of the TECC acquisition curve. Similar to male mice, the averaged S4-S6 terminal sessions were also significantly different for both CR percentage (Rab10^+/+^= 49.391 ± 14.258; Rab10^+/−^ = 32.804 ± 9.469; *t*_(22)_ = 2.964, *p* = 0.0072, 95% CI [5.64, 31.93]; Fig. 8*B*) and CR amplitude (Rab10^+/+^=0.572 ± 0.165; Rab10^+/−^ = 0.362 ± 0.104; *t*_(22)_ = 2.086, *p* = 0.0488, 95% CI [−0.417, −0.001]; Fig. 8*D*).

**Figure 9.**
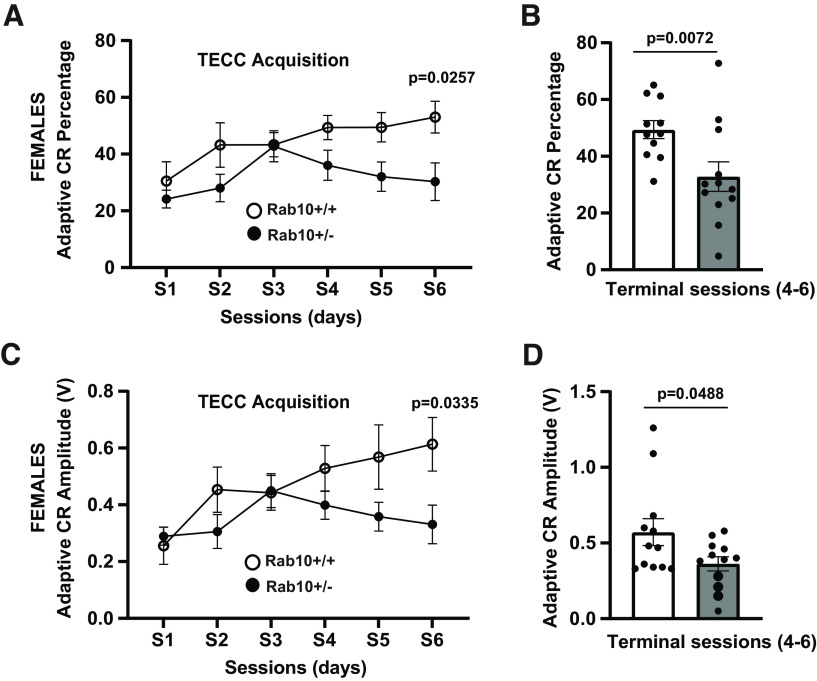
Female Rab10^+/−^ mice exhibit normal sensorimotor performance in TECC paradigm. ***A***, Acquisition curve of unconditioned response (UR) percentage. The number of mice was 11 for Rab10^+/+^ and 12 for Rab10^+/−^ genotype. ***B***, Graph shows that there is no significant difference between genotypes in UR percentage during the terminal sessions (4–6) of the TECC acquisition curve. Error bars indicate SEM. Two-tailed unpaired *t* test, *p* = 0.8931. ***C***, Acquisition curve of unconditioned response (UR) amplitude. The number of mice was 11 for Rab10^+/+^ and 12 for Rab10^+/−^ genotype. ***D***, Graph indicates that female Rab10^+/−^ mice do not exhibit deficit in UR amplitude during the terminal sessions (4–6) of the TECC acquisition curve. Error bars indicate SEM. Two-tailed unpaired *t* test, *p* = 0.5329.

Unconditioned responses were recorded to evaluate whether learning was affected by sensorimotor performance. Similar to male mice, female mice of both genotypes had similar UR percentage (Rab10^+/+^ = 83.270 ± 24.038; Rab10^+/−^ = 82.624 ± 23.851; *t*_(22)_ = 0.136, *p* = 0.893, 95% CI [−10.515, 9.222]; Fig. 9*A*,*B*) and UR amplitude (Rab10^+/+^ = 3.393 ± 0.979; Rab10^+/−^ = 2.919 ± 0.842; *t*_(22)_ = 0.634, *p* = 0.533, 95% CI [−2.031, 1.082]; Fig. 9*C*,*D*).

In summary, these behavioral studies suggest that Rab10 negatively regulates object location memory, whereas it is required for higher-order learning as demonstrated by the TECC test.

## Discussion

Our findings collectively suggest that reducing Rab10 leads to changes in the expression of genes associated with neuroinflammation, aging, and neurotransmission. Behavioral phenotypes of Rab10^+/−^ mice include a sex-dependent improvement in hippocampus-dependent associative learning (OIP test) and a significant impairment in trace eyeblink classical conditioning. These data indicate an opposing role of Rab10 in brain areas involved in contextual versus associative learning.

Identification of genes and pathways that mediate molecular neuroresilience against Alzheimer’s disease may represent promising avenues of therapeutic development. Given that membrane trafficking has been implicated in nearly every aspect of brain function, including neuronal plasticity, maintenance and degeneration, AD risk genes involved in organelle composition, endocytosis, lipid biology and neuroimmune function may represent potential targets for prevention and treatment. Indeed, many AD-associated proteins are involved in intracellular trafficking, and some of them are directly regulated by Rab proteins. As one central hub for membrane trafficking, Rab GTPases have been directly and indirectly implicated in the progression of neurodegeneration (Kiral et al., 2018). This is not surprising, given that neuron-specific Rab-GTPases are highly enriched in synapses and localized on endosomes, suggesting a crucial role of Rab-dependent membrane trafficking in synaptic functions (Chan et al., 2011; Jin et al., 2012). Moreover, in postmortem cholinergic basal forebrain neurons and CA1 pyramidal neurons of sporadic AD patients, several Rab proteins are upregulated, including Rab4, Rab5, Rab6, Rab7, and Rab27, indicating an overactivation of the endocytic machinery (Scheper et al., 2007; Ginsberg et al., 2010, 2011). However, it is unclear whether Rab protein upregulation drives pathology or is a protective compensatory mechanism. Several recent studies focused on Rab10 GTPase as a potential protective factor in AD. A whole genome sequencing identified a rare variant (rs142787485) in the 3′ untranslated region (UTR) of Rab10 gene that reduces Rab10 activity and confers resilience against AD even in elderly individuals with genetic risk for AD (Ridge et al., 2017). Additionally, RNAi screening of Rab proteins in APP processing revealed that silencing several Rab proteins including Rab10 leads to a reduction in amyloid level (Udayar et al., 2013). Moreover, a recent study also implicated Rab10 in AD-associated tau pathology, showing that LRRK2-mediated phosphorylation of Rab10 at Threonine 73 (pRab10-T73) leads to colocalization of pRab10-T73 with pTau (Yan et al., 2018). Additionally, LRRK2-pRab10 signaling controls immunologic response by driving micropinocytosis in phagocytic cells via the PI3K-Akt pathway (Z. Liu et al., 2020). Considering that the genetic variation in LRRK2 enhances susceptibility to the second most common neurodegenerative disorder, Parkinson’s disease (Tolosa et al., 2020), these findings render Rab10 signaling pathways as the common mechanisms of neurodegeneration (Wareham et al., 2022).

In the current study, we have used Rab10-deficient mice to evaluate *in vivo* the contribution of Rab10 to neuronal function, and to identify Rab10 targets that might be the molecular mediators of resilience against neurodegeneration. Differentially expressed genes in the Rab10-deficient mouse brain are associated with neuroinflammation (Vegfa, Galc) and neuron-glia interaction (Rela); neuroplasticity, neurodevelopment, and aging (Vegfa, Phf21a, Rela, Prkaa2, Emcn, Atm); metabolism (Stx2, Lsr, Atp6v0d1, Galc, Rela, Prkaa2, Ppp2r5e); compartmentalization and structural integrity (Stx2, Atp6v0d1, Syt4, Grin2d, Stx1b); and neurotransmission (Stx2, Atp6v0d1, Syt4, Rela. Grin2d, Ppp2r5e, Stx1b). Among the downregulated genes, a potential therapeutical target is the vascular endothelial growth factor (VEGF) signaling protein expressed by endothelial cells and neurons for cell growth and maintenance. Although an increasing number of studies associate VEGF proteins with clinical manifestation of AD, the role of these proteins in neurodegeneration is complex because of the diverse signaling pathways that are VEGF dependent. Several recent studies indicate that upregulation of VEGF is associated with the dysfunction of the blood brain barrier, more severe tau pathology and accelerated cognitive decline (T. Thomas et al., 2015; Mahoney et al., 2021; Ali et al., 2022). In addition, Endomucin (Emcn), an endothelial marker and modulator of VEGF signaling (Park-Windhol et al., 2017), was also downregulated in the cortex of Rab10-deficient mice.

Interestingly, the genes upregulated in Rab10-deficient mice are primarily associated with neuroplasticity, neurotransmission, as well as compartmentalization and structural integrity. It was reported that the overall level of Syt4 is downregulated in the brain of AD model mice (Parra-Damas et al., 2014). However, in dystrophic axons surrounding amyloid plaques, Syt4 is upregulated as an attempt at regenerative sprouting by damaged axons (Tratnjek et al., 2013; Parra-Damas et al., 2014).

Another gene in the Rab10^+/−^ brain is PRKAA2, which encodes the AMPKα2 isoform. As a master kinase of energy metabolism homeostasis, AMPK has been associated with cellular dysfunctions observed in AD (Wang et al., 2019). Surprisingly, AMPKα2 is reduced in the hippocampus in sporadic AD, but not in familial AD, while the AMPKα1 isoform is upregulated in both forms of AD and in murine models of the disease (Zimmermann et al., 2020).

Another surprising DEG upregulated in the Rab-deficient mice is Grin2d, which encodes glutamate ionotropic receptor NMDA type subunit 2D (GluN2D/GRIN2D). Recent studies have shown that Grin2d genetic variants are linked to developmental and early infantile epileptic encephalopathy (Li et al., 2016; XiangWei et al., 2019). Recently identified variants were shown to reduce the surface-to-total protein level ratio, indicating a deficit in the trafficking of GluN2D subunit (XiangWei et al., 2019). Interestingly, treatment with memantine was shown to ameliorate seizure in these patients (XiangWei et al., 2019). Memantine is an Alzheimer’s disease drug that strongly impacts cognitive function by preferentially inhibiting GluN2C-containing and/or GluN2D-containing NMDARs (Kotermanski and Johnson, 2009; Kotermanski et al., 2009). Whether abnormal trafficking of GluN2D occurs in AD and if this is regulated by Rab10 has not been addressed yet.

Next, we examined whether Rab10-deficiency impacts behavior. We showed that reducing Rab10 level enhances spatial recognition memory performance, in the OIP task where the subject associates an object with the place in which it was previously presented. OIP measures hippocampus-dependent spatial memory and depends on the interaction between the hippocampus, perirhinal cortex and the medial prefrontal cortex (Denninger et al., 2018). OIP memory requires activation of the hippocampal glutamatergic receptor transmission (Barker and Warburton, 2015). It has been shown that OIP performance is sex dependent (Cost et al., 2012), which is consistent with our result showing improved OIP performance in male Rab10-deficient mice, but not in females. The signaling pathways mediating sex dependent OIP performance are yet to be identified. It is important to note that OIP memory deficit occurs in AD patients (Fowler et al., 2002). Thus, our finding that reducing Rab10 enhances OIP performance provides the behavioral evidence for Rab10-mediated neuroresilience. Although the precise molecular mechanism of Rab10-dependent inhibition of spatial learning is unknown, we propose that the molecular mechanism involves downregulation of signaling pathways that control neurotransmission, neuronal metabolism, and neuroplasticity.

Contrary to OIP, behavioral performance in trace eyeblink classical conditioning was negatively affected by Rab10 deficiency. TECC is a higher-order procedure that has common features with declarative memory formation in humans. Similar to simple eyeblink conditioning, TECC relies on intact cerebellar-brainstem circuitry, but also requires network interdependency across multiple higher-level brain structures, including the dorsal hippocampus and the cortex (Cheng et al., 2008). It has been established that the memory of the acquired trace eyeblink-conditioned responses is localized in the primary motor cortex (M1) and involves NMDA-specific glutamate receptor function (Hasan et al., 2013). Notably, deletion of the *grin1* gene (encoding GluN1 subunit), specifically in the M1 cortex, impairs performance in TECC (Hasan et al., 2013). In contrast, *grin1* deletion in the hippocampal CA3 only does not interfere with TECC memory formation (Kishimoto et al., 2006). Given that the NMDA receptors of the dorsal hippocampus are critical for adaptive CR timing memory, and lesions of the hippocampus impair TECC (Kishimoto et al., 2006), it has been proposed that the dorsal hippocampus and the cortex play different roles in TECC memory. In this model, the dorsal hippocampus organizes spatial computations, while the M1 cortex performs spatial memory and temporal associations, as well as acquisition and storage (Hasan et al., 2013). Thus, Rab10-dependent TECC performance indicates that Rab10 is involved in cortical cell assemblies to mediate declarative memory, while it acts as a negative regulator of spatial memory, highly dependent on the dorsal hippocampus. Future studies such as spatial gene expression and spatial proteomics are needed to elucidate the downstream targets of Rab10 signaling that mediate differential roles of this protein in behavioral paradigms.

Based on the data presented here, we conclude that Rab10-deficient mice can be used to better understand the role of Rab10 in brain function, and that crossing these mice with mouse models of AD can provide insights into the molecular and cellular mechanisms underlying Rab10-dependent molecular neuroresilience.

## Synthesis

Reviewing Editor: Karl Herrup, University of Pittsburgh School of Medicine Decisions are customarily a result of the Reviewing Editor and the peer reviewers coming together and discussing their recommendations until a consensus is reached. When revisions are invited, a fact-based synthesis statement explaining their decision and outlining what is needed to prepare a revision will be listed below. The following reviewer(s) agreed to reveal their identity: NONE.

The authors use heterozygous Rab10 knockout mice to perform cellular, molecular, and behavioral assessments of the effects of a reduced Rab10 level in mouse brain. They show that Rab10 deficiency is correlated with spatial memory and higher-order learning defects. The authors perform transcriptome profiling and find that Rab10 downregulation is associated with activation of signaling pathways that are involved with neuronal metabolism, structural integrity, neurotransmission, and neuroplasticity. At the same time, Rab10 reduction was correlated with reduced activation of neuroinflammatory and aging pathways. Despite increasing evidence that Rab10 serves as a neuroprotective factor for Alzheimer’s and Parkinson’s disease, the mechanism behind the apparent protection remains mysterious. The Rab10-deficient mice described in this paper are a potentially important tool to help solve this mystery. While the basics of the work are sound, there are several changes that would considerably strengthen it.

One general concern is that the text overstates the strength of the conclusion. Thus, the authors show that Rab10 reduction leads to selective deficits. While the data are sound, they are not sufficient to conclude that Rab10 “controls” these behaviors. The bioinformatics led them to look at glutamatergic signaling, but the conclusion that GRIN2D mediates the Rab10 effect, while consistent with the data, is not proven by them. Throughout the work, the authors would be well advised to temper their language and acknowledge that the correlations that they see support their hypotheses, but further work is needed to definitively prove their points.

Other statements in the text need attention. In the Abstract the authors state that “Downregulation and inhibition of Rab10 retain normal cognitive function and prevent dementia (“cognitive resilience”).” If this sentence referring to published literature, please list the citation and explain in what context this has been shown (e.g., which animal model). Moving this to the Introduction might be a better solution.

In the Introduction, four genes are listed as having “rare” variants associated with Alzheimer’s disease (AD) risk. This applies to APP, PLD3 and TREM2, but APOE does not belong in this list.

In their behavioral assays the authors conclude that “... reducing Rab10 expression has no effect on motor activity, anxiety-like behavior and working memory in mice.” This is a more detailed example of the problem raised above. The authors perform a limited number of tests so that this conclusion is too strong. Other behavioral tests could reveal alterations in motor activity, anxiety-like behavior, or working memory.

The authors conclude that there is a sex-dependent effect of Rab10 signaling on spatial recognition memory performance.” The data supporting this are weak; the difference between male and female mice may well be due to the difference in the number of animals tested? The number of n for male mice is not indicated in the text. According to the figure legend the number of male mice tested was 13 and 20. The number of female mice tested was 6 and 9. Observing a significant difference for male mice but not female mice does not demonstrate a significant difference between the sexes.

“To evaluate if Rab10 is involved in plasticity in neurons from these brain areas [cerebellum, cortex, and hippocampus], we performed TECC...” Again, this is a serious overstatement. To evaluate whether Rab10 in these brain regions caused the TECC changes, Rab10 would need to be specifically depleted in those regions. Because Rab10 is knocked out in all brain regions, the authors cannot exclude the possibility that the depletion of Rab10 in other brain regions is responsible for the behavioral effect observed.

For the analysis of the acquisition of adaptive conditioned response, the authors combine the results of male and female mice. It would be interesting to see the results separated by sex.

The enormous variability in many of assays is striking. The gene expression data (e.g., Figure 4) is especially odd in this regard. Some discussion of the reason for the variation would be helpful.

Related to this, eNeuro strongly encourages the use of estimation statistics to describe quantitative data such as these. The authors are referred to this editorial for an entrée into why: https://www.eneuro.org/content/8/2/ENEURO.0091-21.2021. Please consider replotting the quantitative data with this type of analysis in mind.

Minor suggestions:

In figure 1B, below the blot “1.0” and “0.5” are indicated. This is the percent reduction from 4 independent experiments. I suggest including a graph plotting all the values. Alternatively, add “% of Rab10” below the blot.

In figure 1A the legend includes the phrase “Rab10 conditional mouse line with CMV-Cre mouse line”. Most likely the authors mean “Rab10 conditional heterozygous mouse”? The phrase should be modified for clarity.
